# The Treatment of Exophthalmic Goitre

**Published:** 1906-09-29

**Authors:** 


### Hospital Clinics.

### THE TREATMENT OF EXOPHTHALMIC GOITRE.

While it would seem from Professor Kocher's results that one more disease is about to be transferred from the physician to the surgeon for curative treatment, there is at present, nevertheless, a general consensus of professional opinion that cases of exophthalmic goitre should not be too hurriedly operated upon. The hygienic, dietetic, and medicinal methods of treatment deserve and repay proper attention bestowed upon them, not only because they may place the sufferer in a condition which better enables him to withstand successfully the shock of operation, should operation be finally resorted to, but also because medical treatment of itself may cure the disease. Graves' Disease is, as a rule, marked by chronicity, the usual duration of the illness being from three to seven or even ten years. And although during this long period exacerbations and complications may occur to harass the patient and to tax the resources of the medical attendant, yet, on the whole, ample time is afforded for a thorough, systematic, and often successful trial of medical measures. The patient should be encouraged in his endeavours by being told of the tendency of the disease to undergo slow cure. At the same time he should be warned that it may be necessary for him to lay aside, from time to time, the active duties of his life, in order that he may obtain the absolute rest of body and mind which is necessary to prevent the malady crossing the boundary-line between safety and danger. Exophthalmic goitre, affecting, as it does, the action of the heart, and interfering with metabolism to the extent of frequently inducing considerable or even excessive emaciation, necessitates bodily rest to lighten the work thrown upon the heart and judiciously forced feeding to cope with the prodigal tissue waste. Concerning rest we may lay down as a practical working rule that those cases should be kept in bed where the pulse rate exceeds 110 per minute while the patient is in the recumbent position. When the digestive system does not resent a full and generous diet, forced feeding should be cautiously pushed; but the tendency to irritative dyspepsia characteristic of the disease demands a constant supervision of the dietary by the physician. Mental rest is quite as important as bodily rest. Whatever be the immediate cause of the disease, whether mental strain or not, there is no doubt that nearly all sufferers from Grave's Disease learn to dread periods of nerve strain and stress because of the subsequent aggravation of their symptoms. For this reason it has been urged that seclusion should be combined with the forced feeding, after the Weir-Mitchell pattern.

Medicinally the drug which has most successfully stood the test of time is belladonna. Given in doses sufficiently large to produce its physiological effects, belladonna may be relied upon to bring about a marked amelioration in the symptoms. Its action is attributed to a reduction in the thyroid secretion. With belladonna may be combined a cardiac tonic, the favourite being strophanthus, in doses of from 8 to 10 minims. If diarrhœa is a feature of the case, belladonna is, of course, inadmissible, and we must content ourselves with the bromides.

Of the legion of remedies which from time to time have been suggested for the cure of this obstinate malady, most have only to be mentioned to receive condemnation. Iron, ergot, opium, and thyroid feeding should be avoided. From iodine, on the other hand, benefit occasionally accrues. There is some conflict of opinion on the effect of this remedy. If the current theory of the pathogenesis of exophthalmic goitre be correct, one would expect that iodine, which stimulates the thyroid, would prove harmful in a disease which owed its presence to an increased thyroid secretion; and, in point of fact, Trousseau found that the use of the iodides tended to precipitate acute paroxysms of the disease. On the other hand, Murray, of Newcastle, and Kocher, men whose opinions in anything regarding goitre are worthy of every respect, found that the inunction of the red iodide of mercury ointment was of great service both in Graves' Disease and in simple goitre. The practical outcome of this clashing of opinion is that iodine should be cautiously administered, always in the form of inunction, and that it should at once be stopped on the appearance of untoward symptoms.

Cold, in the form of the ice-bag applied over the thyroid gland, or precordium, for several hours daily is a simple and efficacious adjunct to the general dietetic and drug treatment. In many cases benefit is at once experienced from its employment. Electricity also still holds a place in the treatment. It probably acts by stimulating the vagus, and so slowing the action of the heart. A weak constant current is applied to both sides of the neck simultaneously for about ten or fifteen minutes daily.

### Operative Treatment.

For the introduction and perfection of the operative treatment we are indebted to Professor Kocher, of Berne. Dividing cases of exophthalmic goitre into three classes, Kocher's published results, as they affect the three classes, are as follows: (1) *Struma vasculosa.* Early Grave's Disease, where goitre, tachycardia, and tremor are present, but exophthalmos absent. Of ten cases of this kind operated on all were cured. (2) *Struma Gravesiana colloides.*—Graves' Disease superadded upon simple goitre. This variety is uncommon in England, but may occur more frequently in Switzerland, where simple goitre is very common. Of this class sixty persons were operated upon, forty-nine were cured, and two greatly improved. (3) *Typical Graves' Disease,* fully established. Of these 106 underwent operation, sixty-two were cured, nine greatly improved, seventeen improved, and nine died. The total mortality was

5 per cent, the mortality in the third class about 5.5 per cent. The operation is still on trial, for these figures are obviously open to varying interpretation. But the statistics clearly show that the earlier a case is operated upon the less the risk to life from the operation and the greater the likelihood of complete cure.

Kocher recommends that all cases should be submitted to careful medical treatment for several weeks previously to being operated upon. He prefers local to general anæsthesia. He finds that the chief danger manifests itself within the first few days after operation. It consists in an exacerbation of the previous symptoms, and may be so severe as to cause death. Once this period is over, the patient may be considered safe. There can be no doubt that the operation is a valuable addition to our methods of dealing with the disease. Practically speaking, we should take each case on its own merits. Thus, an early case which shows signs of rapid advancement should be promptly operated upon; and when a patient is disinclined to submit to a prolonged and distracting course of medical treatment, operation may be offered as a short cut out of the difficulty—a short cut not altogether free from peril, it is true, but, on the whole, presenting a fair likelihood of rapid and lasting cure. In the severest cases the surgeon would be wise to abstain from active interference until medical measures had resulted in an amelioration of the patient's condition, and then to operate, as Kocher advises, in stages, beginning with the ligature first of one and later of another thyroid artery, and finally, after some weeks, when the more urgent symptoms have quieted down, completing the operation by the removal of one lobe of the gland. Operating in this manner, Kocher was able to deal safely and successfully with even the most unpromising cases.

